# Supplementary material for: The Evolution and Characterization of the RNA Interference Pathways in Lophotrochozoa
Source: Genome Biol Evol. 2024 May 7;16(5):evae098. doi: 10.1093/gbe/evae098 (PMC11114477; doi:10.1093/gbe/evae098)
Supplement: evae098_Supplementary_Data [file evae098_supplementary_data.zip › Supplementary_materials.zip!/selected_miRNAs/NoGe_selected_miRNAs/mirdeep_pdf_files/NMRB01000470.1_Notospermus_geniculatus_strain_Ushimado_scaffold470,_whole_genome_shotgun_sequence_14113.pdf]

Provisional ID : NMRB01000470.1_Notospermus_geniculatus_strain_Ushimado_scaffold470,_whole_genome_shotgun_sequence_14113
Score total : 29200.6
Score for star read(s) : 3.9
Score for read counts : 29192.2
Score for mfe : 1.5
Score for randfold : 0
Score for cons. seed : 3
Total read count : 57271
Mature read count : 57255
Loop read count : 0
Star read count : 16

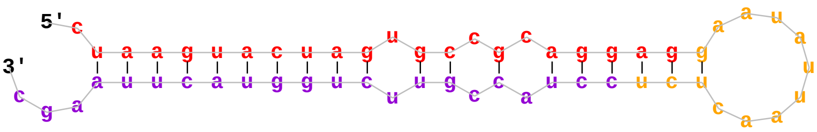

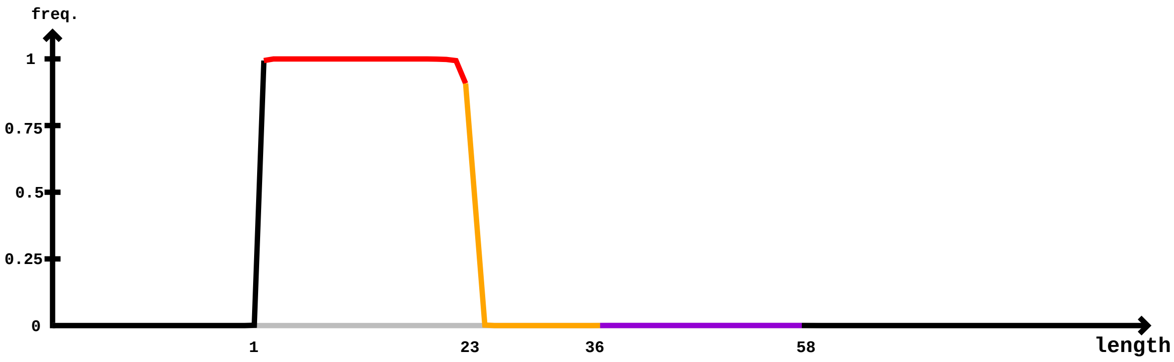

Mature Star

```
5'- augagagccaucgucagauacuaaguacuagugccgcaggaggaauauuaacucuccuaccguucugguacuuaagcuaggacauggcgaacacaucucuggagggagcuga -3'  obs
    augagagccaucgucagauacuaaguacuagugccgcaggaggaauauuaacucuccuaccguucugguacuuaagcuaggacauggcgaacacaucucuggagggagcuga       exp
    ......(((((.(((......((((((((((.((.(.((((((.........)))))).).)).))))))))))......)))))))).....((.((((....)))).)).     reads   mm   sample
    ...................acuaaguacuagugccgcagg........................................................................     2       0    Mm3
    ...................acuaaguacuagugccgcagga.......................................................................     2       0    Mm3
    ...................acuaaguacuagugccgcaggaU......................................................................     1       1    Mm3
    ...................acuaaguacuagugccgcaggag......................................................................     1       0    Mm3
    ...................Ccuaaguacuagugccgcaggag......................................................................     1       1    Mm3
    ...................acuaaguacuagugccgcaggagU.....................................................................     1       1    Mm3
    ....................cuaaguacuagugccgca..........................................................................     1       0    Mm3
    ....................cuaaguacuagugccgcag.........................................................................     8       0    Mm3
    ....................cuaaguacuagugccgUagg........................................................................     1       1    Mm3
    ....................cuaaguacuagugccgcagU........................................................................     1       1    Mm3
    ....................cuaaAuacuagugccgcagg........................................................................     1       1    Mm3
    ....................cuaaguacuagugccgcagg........................................................................     51      0    Mm3
    ....................Nuaaguacuagugccgcagga.......................................................................     1       1    Mm3
    ....................cuaaguacuagugccgcUgga.......................................................................     2       1    Mm3
    ....................Uuaaguacuagugccgcagga.......................................................................     1       1    Mm3
    ....................cuaaguacuagugccgcCgga.......................................................................     2       1    Mm3
    ....................cuaaguacuaguAccgcagga.......................................................................     1       1    Mm3
    ....................cuaaguacuagugccgcaggC.......................................................................     2       1    Mm3
    ....................cuaaguacuagugccgGagga.......................................................................     1       1    Mm3
    ....................cuaaguacuaAugccgcagga.......................................................................     1       1    Mm3
    ....................cuaaguacuagugccgcaggG.......................................................................     3       1    Mm3
    ....................cuaaguacuagugccgcagga.......................................................................     590     0    Mm3
    ....................cuaaguacuaUugccgcagga.......................................................................     1       1    Mm3
    ....................cuaaguacuagugccgcaggU.......................................................................     13      1    Mm3
    ....................cuaaAuacuagugccgcagga.......................................................................     1       1    Mm3
    ....................cuaaguacuaAugccgcaggag......................................................................     5       1    Mm3
    ....................cuaaguGcuagugccgcaggag......................................................................     1       1    Mm3
    ....................cuCaguacuagugccgcaggag......................................................................     1       1    Mm3
    ....................cuaaguacuagugccgcagAag......................................................................     3       1    Mm3
    ....................cuaaguacuagugccgGaggag......................................................................     5       1    Mm3
    ....................cuaaguacuagugccgAaggag......................................................................     29      1    Mm3
    ....................cuaaguacuagugccgcaggUg......................................................................     1       1    Mm3
    ....................cuaaguacuagugccgcUggag......................................................................     20      1    Mm3
```

```
                    Mature                        Star
augagagccaucgucagauacuaaguacuagugccgcaggaggaauauuaacucuccuaccguucugguacuuaagcuaggacauggcgaacacaucucuggagggagcuga
....................cuaaguacuaCugccgcaggag......................................................................   4      1   Mm3
....................cuaaguacuagugUcgcaggag......................................................................   2      1   Mm3
....................cuaaguacuagugccgcaggGg......................................................................   4      1   Mm3
....................cuaGguacuagugccgcaggag......................................................................   3      1   Mm3
....................cuaaguacuagugccAcaggag......................................................................   1      1   Mm3
....................cuaaguacuUgugccgcaggag......................................................................   2      1   Mm3
....................cuaaguacuagugccgcaAgag......................................................................   1      1   Mm3
....................cuaaguacuagugGcgcaggag......................................................................   1      1   Mm3
....................cuUaguacuagugccgcaggag......................................................................   1      1   Mm3
....................cuaaguaAuagugccgcaggag......................................................................   1      1   Mm3
....................cuaaguacuagugccgcCggag......................................................................   16     1   Mm3
....................cuaagAacuagugccgcaggag......................................................................   2      1   Mm3
....................cuaaguaUuagugccgcaggag......................................................................   2      1   Mm3
....................cuaaguacuagugccgcGggag......................................................................   16     1   Mm3
....................cuaaguacuagugccgcaggag......................................................................   6598   0   Mm3
....................cuaaguUcuagugccgcaggag......................................................................   1      1   Mm3
....................cuaaguacuagugccgcaggaU......................................................................   61     1   Mm3
....................cuaaguacuagugccgUaggag......................................................................   14     1   Mm3
....................cuaaguacuaUugccgcaggag......................................................................   7      1   Mm3
....................cuaaguacuagugccgcaggaC......................................................................   69     1   Mm3
....................cuaaguacuagugccgcaggCg......................................................................   3      1   Mm3
....................cAaaguacuagugccgcaggag......................................................................   5      1   Mm3
....................cuaaguacuaguCccgcaggag......................................................................   1      1   Mm3
....................cuaaguacuagAgccgcaggag......................................................................   1      1   Mm3
....................cuaaguacuagugccgcaggaA......................................................................   10     1   Mm3
....................cuaaguacAagugccgcaggag......................................................................   1      1   Mm3
....................cuaagCacuagugccgcaggag......................................................................   1      1   Mm3
....................cuaCguacuagugccgcaggag......................................................................   1      1   Mm3
....................Uuaaguacuagugccgcaggag......................................................................   13     1   Mm3
....................cuGaguacuagugccgcaggag......................................................................   6      1   Mm3
....................Nuaaguacuagugccgcaggag......................................................................   6      1   Mm3
....................cuaaguacuagugccgcCggagg.....................................................................   1      1   Mm3
....................Nuaaguacuagugccgcaggagg.....................................................................   1      1   Mm3
....................cuaaguacuagugccgcaggagU.....................................................................   4034   1   Mm3
....................cuaaguacuagugccgcaggagC.....................................................................   2      1   Mm3
....................cuaaguacuagugccgcaggagA.....................................................................   180    1   Mm3
....................cuaaguacuagugccgcaggagg.....................................................................   76     0   Mm3
....................cuaaguacuagugccgcaggagAa....................................................................   3      1   Mm3
....................cuaaguacuagugccgcaggaggU....................................................................   16     1   Mm3
.....................uaaguacuagugccgcagg........................................................................   2      0   Mm3
.....................uaaguacuagugccgcagga.......................................................................   8      0   Mm3
.....................uaaguacuagugccgcaAga.......................................................................   1      1   Mm3
.....................uaaguacuagugccgcaUgag......................................................................   1      1   Mm3
.....................uaaguacuagugccgcaAgag......................................................................   1      1   Mm3
.....................uaaguacuagugccgcaggag......................................................................   37     0   Mm3
.....................uaaguacuagugccgcaCgag......................................................................   1      1   Mm3
.....................uaaguacuagugccgcaggaU......................................................................   1      1   Mm3
.....................uaaguacuagugccgcaggaC......................................................................   1      1   Mm3
.....................uaaguacuagAgccgcaggag......................................................................   1      1   Mm3
.....................uaaguacuagugccgcaggagU.....................................................................   20     1   Mm3
.....................uaaguacuagugccgcaggagg.....................................................................   4      0   Mm3
.....................uaaguacuagugccgcaggagC.....................................................................   1      1   Mm3
.....................uaaguacuagugccgcaggaggU....................................................................   1      1   Mm3
.......................................................ccuaccguucugguacuuaag....................................   1      0   Mm3

...................acuaaguacuagugccgcagga.......................................................................   10     0   Fm3
...................acuaaguacuagugccgcaggaU......................................................................   1      1   Fm3
....................cuaaguacuagugccgca..........................................................................   11     0   Fm3
....................cuaaguacuagugccgUa..........................................................................   2      1   Fm3
....................cuaaguacuagugccgcag.........................................................................   21     0   Fm3
....................cuaaguacuagugccgcaU.........................................................................   7      1   Fm3
....................cuaaguacuagugccgcaA.........................................................................   3      1   Fm3
....................cuaaguacuagugccgcagU........................................................................   5      1   Fm3
....................cuaaguacuagugccgAagg........................................................................   1      1   Fm3
....................cuaaguacuagugccgcagA........................................................................   1      1   Fm3
....................cuaaguacuagugccgcagg........................................................................   50     0   Fm3
....................cuaaguacuGgugccgcagga.......................................................................   2      1   Fm3
....................cCaaguacuagugccgcagga.......................................................................   1      1   Fm3
....................cuaaguacuagugccgcUgga.......................................................................   4      1   Fm3
....................cuaaguacuaAugccgcagga.......................................................................   2      1   Fm3
```

Mature Star

augagagccaucgucagauacuaaguacuagugccgcaggaggaauauuaacucuccuaccguucugguacuuaagcuaggacauggcgaacacaucucuggagggagcuga			
....................Auaaguacuagugccgcagga...	1	1	Fm3
....................cuaaguacuagugccgGagga...	2	1	Fm3
....................cuaaguacuagugccgcagUa...	1	1	Fm3
....................cuaaguacuagugcUgcagga...	1	1	Fm3
....................cuaaguacuagugccgcaggU...	10	1	Fm3
....................cuaUguacuagugccgcagga...	1	1	Fm3
....................cuaaguacuagugccgAagga...	11	1	Fm3
....................cuaaguacuagugccgUagga...	1	1	Fm3
....................cAaaguacuagugccgcagga...	1	1	Fm3
....................cuaaguacuagugccgcCgga...	3	1	Fm3
....................cuaagCacuagugccgcagga...	1	1	Fm3
....................cuaaguacuagugccgcGgga...	4	1	Fm3
....................cuaaguacuaCugccgcagga...	1	1	Fm3
....................cuaaguacuagugUcgcagga...	4	1	Fm3
....................cuaagNacuagugccgcagga...	1	1	Fm3
....................cuaaguacuagugccgcagga...	1207	0	Fm3
....................cuaaguacuaUugccgcagga...	2	1	Fm3
....................cuaagAacuagugccgcaggag..	1	1	Fm3
....................cuaaguacuaAugccgcaggag..	9	1	Fm3
....................cuaaguacuagugccgAaggag..	27	1	Fm3
....................cuaagNacuagugccgcaggag..	4	1	Fm3
....................cuaaguaUuagugccgcaggag..	1	1	Fm3
....................cuaagCacuagugccgcaggag..	3	1	Fm3
....................cuaaguacuagugccgGaggag..	6	1	Fm3
....................cuaaguacuagugUcgcaggag..	2	1	Fm3
....................cuCaguacuagugccgcaggag..	2	1	Fm3
....................cuaaguacuagugccgcCggag..	27	1	Fm3
....................cuaaguacuagugccgcaggaA..	23	1	Fm3
....................cuUaguacuagugccgcaggag..	1	1	Fm3
....................Uuaaguacuagugccgcaggag..	5	1	Fm3
....................cuaaguacuagugccgcaCgag..	2	1	Fm3
....................cuaaguacuagugccgcagUag..	2	1	Fm3
....................cGaaguacuagugccgcaggag..	2	1	Fm3
....................cuaaguacuagugAcgcaggag..	2	1	Fm3
....................cuaaguacuagugccgcaggag..	7600	0	Fm3
....................cCaaguacuagugccgcaggag..	3	1	Fm3
....................cuaaguacuagugccgcagAag..	1	1	Fm3
....................cuaaguacuagugccgcaNgag..	1	1	Fm3
....................Nuaaguacuagugccgcaggag..	6	1	Fm3
....................cuaaguacuagugcGgcaggag..	1	1	Fm3
....................cuaaguacuagugccgcaggUg..	1	1	Fm3
....................cuaaguacuGgugccgcaggag..	1	1	Fm3
....................cuaaguacuagugcUgcaggag..	5	1	Fm3
....................cuaaguacuagugccgcUggag..	26	1	Fm3
....................cuaUguacuagugccgcaggag..	1	1	Fm3
....................cuaaguUcuagugccgcaggag..	1	1	Fm3
....................cuaaguacuagugccCcaggag..	1	1	Fm3
....................cuaaguacuagugccgcaggaU..	67	1	Fm3
....................cuaCguacuagugccgcaggag..	1	1	Fm3
....................cuaaguacuaUugccgcaggag..	14	1	Fm3
....................cuaaguacuagugccgUaggag..	18	1	Fm3
....................cAaaguacuagugccgcaggag..	4	1	Fm3
....................Auaaguacuagugccgcaggag..	5	1	Fm3
....................cuGaguacuagugccgcaggag..	2	1	Fm3
....................cuaaguacuagugccUcaggag..	1	1	Fm3
....................cuaaguacuaCugccgcaggag..	6	1	Fm3
....................cuaaguacuagugccgcaggaC..	117	1	Fm3
....................cuaagGacuagugccgcaggag..	1	1	Fm3
....................cuaaguacuagugccgcGggag..	23	1	Fm3
....................cuaaguGcuagugccgcaggag..	1	1	Fm3
....................cuaaguaGuagugccgcaggag..	1	1	Fm3
....................cuaaguacuagugccgAaggagg...	1	1	Fm3
....................cuaaguacuaAugccgcaggagg...	1	1	Fm3
....................cuaaguacuagugccgcaggagA...	363	1	Fm3
....................cuaaguacuagugccgcaggagg...	143	0	Fm3
....................cuaaguacuagugccgcCggagg...	1	1	Fm3
....................cuGaguacuagugccgcaggagg...	1	1	Fm3
....................cuaaguacuagugccgUaggagg...	1	1	Fm3
....................cuaaguacuagugccgcaggagC...	1	1	Fm3
....................cuaaguacuagugccgcaggagU...	2028	1	Fm3

Mature **Star**

```
augagagccaucgucagauacuaaguacuagugccgcaggaggaaauuaacucuccuaccguucugguacuuaagcuaggacauggcgaacacaucucuggagggagcuga
....................cuaaguacuagugccgcaggagUa...................................................................   3     1   Fm3
....................cuaaguacuaguCccgcaggagga...................................................................   1     1   Fm3
....................cuaaguacuagugccgcaggaggU...................................................................   9     1   Fm3
....................cuaaguacuagugccgcaggagAa...................................................................   13    1   Fm3
.....................uaaguacuagugccgcag........................................................................   3     0   Fm3
.....................uaaguacuagugccgcagg.......................................................................   1     0   Fm3
.....................uaaguacuagugcGgcagga......................................................................   1     1   Fm3
.....................uaaguacuagugccgcagga......................................................................   8     0   Fm3
.....................uaaguacuagugccgcCggag.....................................................................   1     1   Fm3
.....................uaaguacuagugccgcaggaU.....................................................................   3     1   Fm3
.....................uaaguacuagugccgcaggaC.....................................................................   1     1   Fm3
.....................uaaguacuagugccgcaggag.....................................................................   37    0   Fm3
.....................uaaguacuagugccgcaggCg.....................................................................   1     1   Fm3
.....................uaaguacuagugccgcaggagU....................................................................   13    1   Fm3
.....................uaaguacuagugccgcaggagg....................................................................   7     0   Fm3
.....................uaaguacuagugccgcaAgagg....................................................................   1     1   Fm3
.....................uGaguacuagugccgcaggagg....................................................................   3     1   Fm3
.....................uaaguacuagugccgcaggagga...................................................................   5     0   Fm3
........................guacuagugccgcaggag.....................................................................   2     0   Fm3
......................................................ccuaccguucugguacuuaagc...................................   5     0   Fm3
......................................................ccuaccguuAugguacuuaagc...................................   1     1   Fm3
.......................................................cuaccguucugguacuuaagcu..................................   3     0   Fm3

...................acuaaguacuagugccgcagga......................................................................   1     0   Mm1
....................cuaaguacuagugccgca.........................................................................   5     0   Mm1
....................cuaaguacuagugccgcag........................................................................   9     0   Mm1
....................cuaaguacuagugccgcagg.......................................................................   1     0   Mm1
....................cuaaguacuagugccgcagU.......................................................................   9     1   Mm1
....................cuaaguacuagugccgcaggU......................................................................   14    1   Mm1
....................cuaaguacuagugccgAagga......................................................................   2     1   Mm1
....................cuaaguUcuagugccgcagga......................................................................   1     1   Mm1
....................cuaaguacuagugccgcGgga......................................................................   1     1   Mm1
....................cuaaguacuagugccgcCgga......................................................................   3     1   Mm1
....................cuaaguacuagugccgcagga......................................................................   293   0   Mm1
....................cuaaguacuagugccgcUgga......................................................................   1     1   Mm1
....................cuaaguacuaUugccgcagga......................................................................   1     1   Mm1
....................cuaagAacuagugccgcaggag.....................................................................   1     1   Mm1
....................cuaaguUcuagugccgcaggag.....................................................................   1     1   Mm1
....................cuaaguacuagugccgcaggaU.....................................................................   11    1   Mm1
....................cuaaguacuagugccgcUggag.....................................................................   9     1   Mm1
....................cuUaguacuagugccgcaggag.....................................................................   1     1   Mm1
....................cuaaguacuaUugccgcaggag.....................................................................   1     1   Mm1
....................Nuaaguacuagugccgcaggag.....................................................................   3     1   Mm1
....................cuaaguCcuagugccgcaggag.....................................................................   1     1   Mm1
....................cuaaguacuagugccgGaggag.....................................................................   2     1   Mm1
....................cuaaguacuagugccgcaggag.....................................................................   2957  0   Mm1
....................cuaaguacuagugccgcaUgag.....................................................................   1     1   Mm1
....................Uuaaguacuagugccgcaggag.....................................................................   5     1   Mm1
....................cuaaguacuagugccgAaggag.....................................................................   13    1   Mm1
....................cuaaguacuagugccgcaggaA.....................................................................   15    1   Mm1
....................cuaaguacuaAugccgcaggag.....................................................................   5     1   Mm1
....................cuaaguacuagugccgcCggag.....................................................................   8     1   Mm1
....................cuaaguacuagugAcgcaggag.....................................................................   1     1   Mm1
....................cuaaguacuagugccgUaggag.....................................................................   6     1   Mm1
....................cuCaguacuagugccgcaggag.....................................................................   1     1   Mm1
....................cuaaguacuagugccgcGggag.....................................................................   14    1   Mm1
....................cAaaguacuagugccgcaggag.....................................................................   1     1   Mm1
....................cuaCguacuagugccgcaggag.....................................................................   2     1   Mm1
....................cuaaguGcuagugccgcaggag.....................................................................   1     1   Mm1
....................cuaaguacuagugccgcaggaC.....................................................................   9     1   Mm1
....................cuaaguacuaCugccgcaggag.....................................................................   2     1   Mm1
....................cCaaguacuagugccgcaggag.....................................................................   1     1   Mm1
....................cuaaguacuagugccgcaggagU....................................................................   5899  1   Mm1
....................cuaaguacuagugccgcaggagA....................................................................   30    1   Mm1
....................cuaaguacuagugccgcaggagC....................................................................   2     1   Mm1
....................cuaaguacuagugccgcaggagg....................................................................   33    0   Mm1
....................cuaaguacuagugccgcaggaggU...................................................................   16    1   Mm1
.....................uaaguacuagugccgcaggag.....................................................................   9     0   Mm1
.....................uaaguacuagugccgcaggagU....................................................................   30    1   Mm1

...................acuaaguacuagugccgcagga......................................................................   5     0   Mm2
```

```
                    Mature                            Star
augagagccaucgucagauacuaaguacuagugccgcaggaggaauauuaacucuccuaccguucugguacuuaagcuaggacauggcgaacacaucucuggagggagcuga
....................cuaaguacuagugccgca..........................................................................    3      0    Mm2
....................cuaaguacuagugccgcaU.........................................................................    3      1    Mm2
....................Uuaaguacuagugccgcagg........................................................................    1      1    Mm2
....................cuaaguacuagugccgcagU........................................................................    2      1    Mm2
....................cuaaguacuagugccgcagg........................................................................    50     0    Mm2
....................cuaaguacuagugccgcagC........................................................................    1      1    Mm2
....................cuaaguacuagugccgAagga.......................................................................    3      1    Mm2
....................cuaaguacuagugccgcUgga.......................................................................    4      1    Mm2
....................cuaaguacuagugccgcaggU.......................................................................    13     1    Mm2
....................cuaaguacuaguCccgcagga.......................................................................    1      1    Mm2
....................cuaaguacuagugccgUagga.......................................................................    2      1    Mm2
....................cuaaguacuagugccgcagga.......................................................................    887    0    Mm2
....................cCaaguacuagugccgcagga.......................................................................    1      1    Mm2
....................cuaaguacuagugccgGagga.......................................................................    2      1    Mm2
....................cAaaguacuagugccgcagga.......................................................................    1      1    Mm2
....................cuaaguacuaCugccgcagga.......................................................................    1      1    Mm2
....................cuaaguUcuagugccgcagga.......................................................................    1      1    Mm2
....................cuaaguacuagugccgcGgga.......................................................................    4      1    Mm2
....................cuaaguacuagugccgcCgga.......................................................................    2      1    Mm2
....................cuaaguacuagugccgcaggG.......................................................................    10     1    Mm2
....................cuaaguacuagugccgAaggag......................................................................    26     1    Mm2
....................cuaaguacuagCgccgcaggag......................................................................    1      1    Mm2
....................cuUaguacuagugccgcaggag......................................................................    1      1    Mm2
....................Uuaaguacuagugccgcaggag......................................................................    11     1    Mm2
....................cuaaguacuaUugccgcaggag......................................................................    6      1    Mm2
....................cuaagCacuagugccgcaggag......................................................................    2      1    Mm2
....................cuaaguacuagugccgGaggag......................................................................    7      1    Mm2
....................cuaaguacuaAugccgcaggag......................................................................    7      1    Mm2
....................cuaaguacuagugccgcaggaC......................................................................    59     1    Mm2
....................cuaUguacuagugccgcaggag......................................................................    2      1    Mm2
....................cuaaguacuagugccgcaggaA......................................................................    3      1    Mm2
....................cuaaguacuagugccgUaggag......................................................................    16     1    Mm2
....................cuaaguacuagugAcgcaggag......................................................................    2      1    Mm2
....................cuaaguacuagugccgcaggag......................................................................    6372   0    Mm2
....................cGaaguacuagugccgcaggag......................................................................    1      1    Mm2
....................cAaaguacuagugccgcaggag......................................................................    1      1    Mm2
....................cuaaguacuagugccgcGggag......................................................................    38     1    Mm2
....................cuaaguacuagugccgcaggaU......................................................................    80     1    Mm2
....................cuaaguacuagugccgcaggUg......................................................................    2      1    Mm2
....................cuaaguacuaCugccgcaggag......................................................................    8      1    Mm2
....................cuaaguacuagugccgcUggag......................................................................    20     1    Mm2
....................cuaCguacuagugccgcaggag......................................................................    1      1    Mm2
....................cuaaguacuUgugccgcaggag......................................................................    2      1    Mm2
....................cuaaguUcuagugccgcaggag......................................................................    4      1    Mm2
....................cuaagAacuagugccgcaggag......................................................................    1      1    Mm2
....................cuaaguacuagugcUgcaggag......................................................................    1      1    Mm2
....................cuaaguacuagugccgcCggag......................................................................    13     1    Mm2
....................cuaaguacuagugccgcaggCg......................................................................    1      1    Mm2
....................cuaaguacuagugccgcaggGg......................................................................    3      1    Mm2
....................Nuaaguacuagugccgcaggag......................................................................    2      1    Mm2
....................cuGaguacuagugccgcaggag......................................................................    2      1    Mm2
....................cuaaguacuagugcGgcaggag......................................................................    2      1    Mm2
....................cuaaguacuagugccgcaUgag......................................................................    1      1    Mm2
....................cuaaguacuagugccgcaggaCg.....................................................................    1      1    Mm2
....................cuaaguacuagugccgcaggagU.....................................................................    1826   1    Mm2
....................cuaaguacuagugccgcaggagg.....................................................................    139    0    Mm2
....................cuaaguacuagugccgcUggagg.....................................................................    1      1    Mm2
....................cuaaguacuaCugccgcaggagg.....................................................................    1      1    Mm2
....................cuaaguacuagugccgUaggagg.....................................................................    1      1    Mm2
....................cuaaguacuagugccgcaggagA.....................................................................    142    1    Mm2
....................cuaaguacuagugccgcaggaggU....................................................................    7      1    Mm2
....................cuaaguacuagugccgcaggagAa....................................................................    1      1    Mm2
.....................uaaguacuagugccgcagga.......................................................................    7      0    Mm2
.....................uaaguacuagugccgcaggaU......................................................................    1      1    Mm2
.....................uaaguacuagugccgcaggag......................................................................    33     0    Mm2
.....................uaaguacuagugccgcaggagg.....................................................................    20     0    Mm2
.....................uaaguacuagugccgcaggagU.....................................................................    21     1    Mm2
.......................aguacuagugccgcaggag......................................................................    1      0    Mm2
.......................................................ccuaccguucugguacuuaagc...................................    1      0    Mm2

....................cuaaguacuagugccgca..........................................................................    6      0    Fm2
```

```
                    Mature                            Star
augagagccaucgucagauacuaaguacuagugccgcaggaggaauauuaacucuccuaccguucugguacuuaagcuaggacauggcgaacacaucucuggagggagcuga
....................cuaaguacuagugccgcaA.........................................................................    5     1   Fm2
....................cuaaguacuCgugccgcagg........................................................................    5     1   Fm2
....................cuaaguacuagugccgcagg........................................................................    32    0   Fm2
....................cuaaguacuagugccgcagUa.......................................................................    1     1   Fm2
....................Nuaaguacuagugccgcagga.......................................................................    1     1   Fm2
....................cuaaguacuUgugccgcagga.......................................................................    1     1   Fm2
....................cuaaguacuagugccgcagga.......................................................................    292   0   Fm2
....................cuaaguacuagugccgcGgga.......................................................................    1     1   Fm2
....................cuaaguacuaUugccgcagga.......................................................................    3     1   Fm2
....................cuaaguacuagugccgcUgga.......................................................................    1     1   Fm2
....................cuaaguacuaCugccgcagga.......................................................................    1     1   Fm2
....................Auaaguacuagugccgcagga.......................................................................    1     1   Fm2
....................cuaaguacuagugccgcCgga.......................................................................    1     1   Fm2
....................cuaaguacuagugccgcaggG.......................................................................    2     1   Fm2
....................cuaaguacuagugccgGaggag......................................................................    2     1   Fm2
....................cuaaguacuagugccgcUggag......................................................................    11    1   Fm2
....................cuaaguNcuagugccgcaggag......................................................................    2     1   Fm2
....................Auaaguacuagugccgcaggag......................................................................    2     1   Fm2
....................cuaaguacuagugccgcagAag......................................................................    1     1   Fm2
....................cuaaguacuaCugccgcaggag......................................................................    4     1   Fm2
....................cuaaguacuagugccgcaggaC......................................................................    44    1   Fm2
....................cuaagCacuagugccgcaggag......................................................................    1     1   Fm2
....................Nuaaguacuagugccgcaggag......................................................................    1     1   Fm2
....................cuaaguacuagugccgUaggag......................................................................    4     1   Fm2
....................cuaaguacuagugccgcaggaA......................................................................    6     1   Fm2
....................cuaaguacuaUugccgcaggag......................................................................    4     1   Fm2
....................cNaaguacuagugccgcaggag......................................................................    1     1   Fm2
....................cuaaguacuagugcUgcaggag......................................................................    2     1   Fm2
....................cuaCguacuagugccgcaggag......................................................................    1     1   Fm2
....................cuaaguacuagugccgcGggag......................................................................    8     1   Fm2
....................cAaaguacuagugccgcaggag......................................................................    1     1   Fm2
....................cuaaguacAagugccgcaggag......................................................................    1     1   Fm2
....................Uuaaguacuagugccgcaggag......................................................................    4     1   Fm2
....................cCaaguacuagugccgcaggag......................................................................    1     1   Fm2
....................cuaaguacuaAugccgcaggag......................................................................    1     1   Fm2
....................cuaaguacuagugccgcCggag......................................................................    8     1   Fm2
....................cuaaguacuagugccgcagUag......................................................................    1     1   Fm2
....................cuaaguacuagugccgcaggaU......................................................................    19    1   Fm2
....................cuaaguacuagugccgAaggag......................................................................    12    1   Fm2
....................cuaaguacuagugccgcaUgag......................................................................    1     1   Fm2
....................cuaaguacuagugccgcaggag......................................................................    3042  0   Fm2
....................cuaaguacuagAgccgcaggag......................................................................    1     1   Fm2
....................cuaaguacuagCgccgcaggag......................................................................    1     1   Fm2
....................cuaaguacuagugccgcaggagg.....................................................................    92    0   Fm2
....................cuaaguacuagugccgcaggagA.....................................................................    42    1   Fm2
....................cuaaguacuagugccgcCggagg.....................................................................    2     1   Fm2
....................cuaaguacuagugccgcaggagU.....................................................................    3900  1   Fm2
....................cuaaguacuagugccgcaggagC.....................................................................    4     1   Fm2
....................cuaaguacuagugccgcaggaggU....................................................................    19    1   Fm2
....................cuaaguacuagugccgcaggaggG....................................................................    1     1   Fm2
.....................uaaguacuagugccgcaggag......................................................................    4     0   Fm2
.....................uaaguacuagugccgcaggagU.....................................................................    10    1   Fm2

...................acuaaguacuagugccgcagga.......................................................................    25    0   Fm1
...................acuaaguacuagugccUcagga.......................................................................    1     1   Fm1
...................acuaaguacuagugccgcaggaU......................................................................    11    1   Fm1
....................cuaaguacuagugccgcU..........................................................................    2     1   Fm1
....................cuaaguacuagugccgca..........................................................................    6     0   Fm1
....................cuaaguacuagugccgcag.........................................................................    6     0   Fm1
....................cuaaguacuagugccgcCg.........................................................................    1     1   Fm1
....................cuaaguacuagugccgcaU.........................................................................    4     1   Fm1
....................cuaaguacuagugccgcagg........................................................................    24    0   Fm1
....................cuaaguacuagugccgGagg........................................................................    1     1   Fm1
....................cuaaguacuagugccgcagU........................................................................    6     1   Fm1
....................cuaaguacuagugccgcUgg........................................................................    1     1   Fm1
....................cuaaguacuagugccgcagga.......................................................................    1331  0   Fm1
....................cuaaguGcuagugccgcagga.......................................................................    1     1   Fm1
....................Nuaaguacuagugccgcagga.......................................................................    1     1   Fm1
....................cuaaguacuaCugccgcagga.......................................................................    1     1   Fm1
....................cuaaguacuagugccgAagga.......................................................................    4     1   Fm1
```

```
                    Mature                                Star
augagagccaucgucagauacuaaguacuagugccgcaggaggaauauuaacucuccuaccguucugguacuuaagcuaggacauggcgaacacaucucuggagggagcuga
....................cuaaguacuagugccgcaggG.......................................................................    7      1    Fm1
....................cuaaguacuagugccgcGgga.......................................................................    7      1    Fm1
....................cuaaguacuagugccAcagga.......................................................................    1      1    Fm1
....................cuaaguacuagugccgcaggU.......................................................................    11     1    Fm1
....................cuaaguacuagugccgcUgga.......................................................................    5      1    Fm1
....................cuaaguacuagugUcgcagga.......................................................................    2      1    Fm1
....................cuGaguacuagugccgcagga.......................................................................    2      1    Fm1
....................cuaaguacuaAugccgcagga.......................................................................    1      1    Fm1
....................Uuaaguacuagugccgcagga.......................................................................    1      1    Fm1
....................cAaaguacuagugccgcagga.......................................................................    3      1    Fm1
....................cuaaguacuagugccgcCgga.......................................................................    1      1    Fm1
....................cuaaguacuagugccgUagga.......................................................................    2      1    Fm1
....................cuaaguacuagugccgGagga.......................................................................    1      1    Fm1
....................cuaaguacuagugccgcaggUg......................................................................    1      1    Fm1
....................cuaaguacuUgugccgcaggag......................................................................    1      1    Fm1
....................cuaaguacuagugccgcaggag......................................................................    3405   0    Fm1
....................cuaaguacuagugccgUaggag......................................................................    3      1    Fm1
....................cuaaguacuaguUccgcaggag......................................................................    1      1    Fm1
....................cuaaguacuagugccgcaUgag......................................................................    1      1    Fm1
....................cuaaguacuagugcUgcaggag......................................................................    2      1    Fm1
....................cuaaguacuagAgccgcaggag......................................................................    1      1    Fm1
....................cuaaguacuaUugccgcaggag......................................................................    4      1    Fm1
....................cuGaguacuagugccgcaggag......................................................................    1      1    Fm1
....................cuaaguacuagugccgcagUag......................................................................    2      1    Fm1
....................cuaaguacuagCgccgcaggag......................................................................    2      1    Fm1
....................cuaaguacuagugccgcUggag......................................................................    9      1    Fm1
....................Uuaaguacuagugccgcaggag......................................................................    5      1    Fm1
....................cAaaguacuagugccgcaggag......................................................................    1      1    Fm1
....................cuaagGacuagugccgcaggag......................................................................    1      1    Fm1
....................cuaaguNcuagugccgcaggag......................................................................    1      1    Fm1
....................cuaaguacuaguAccgcaggag......................................................................    1      1    Fm1
....................cuaaguacuagugccgGaggag......................................................................    4      1    Fm1
....................cuaaguacuagugccgcaggaU......................................................................    87     1    Fm1
....................cuaaguacuagugccgcaggaA......................................................................    3      1    Fm1
....................cuaaguacuagugAcgcaggag......................................................................    1      1    Fm1
....................cuaaguacuagugccgAaggag......................................................................    15     1    Fm1
....................cuaaguacuagugccgcCggag......................................................................    20     1    Fm1
....................Nuaaguacuagugccgcaggag......................................................................    2      1    Fm1
....................cuaaguacuaAugccgcaggag......................................................................    9      1    Fm1
....................cuaaguacuagugccgcGggag......................................................................    13     1    Fm1
....................cuaaguaUuagugccgcaggag......................................................................    2      1    Fm1
....................cCaaguacuagugccgcaggag......................................................................    2      1    Fm1
....................cuaaguacuagugccgcaggaC......................................................................    37     1    Fm1
....................cuaaguacuaCugccgcaggag......................................................................    2      1    Fm1
....................cuaaguUcuagugccgcaggag......................................................................    2      1    Fm1
....................cuaaguacuagGgccgcaggag......................................................................    1      1    Fm1
....................cuaaguGcuagugccgcaggag......................................................................    1      1    Fm1
....................cuaaguacuagugccgcCggagg.....................................................................    2      1    Fm1
....................cuaaguacuagugccgcaggagU.....................................................................    718    1    Fm1
....................cuaaguacuagugccgcaggagg.....................................................................    135    0    Fm1
....................cuaaguacuagugccgcaggaCg.....................................................................    1      1    Fm1
....................cuaaguacuagugccgcaggagC.....................................................................    11     1    Fm1
....................cuaaguacuagugccgcaggagA.....................................................................    219    1    Fm1
....................cuaaguacuagugccgAaggagg.....................................................................    3      1    Fm1
....................cuaaguacuagugccgcaggaggU....................................................................    14     1    Fm1
.....................uaaguacuagugccgcagg........................................................................    2      0    Fm1
.....................uaaguacuagGgccgcagga.......................................................................    1      1    Fm1
.....................uaaguacuagugccgcagga.......................................................................    14     0    Fm1
.....................uaaguacuagugccgcaCga.......................................................................    1      1    Fm1
.....................uaaguacuagugccgcaggag......................................................................    16     0    Fm1
.....................uaaguacuagugccgcCggag......................................................................    1      1    Fm1
.....................uaaguacuagugccgcaggagU.....................................................................    2      1    Fm1
.....................uaaguacuagugccgcaggagg.....................................................................    3      0    Fm1
.......................................................ccuaccguucugguacuuaagc...................................    5      0    Fm1
```
